# PD47 Procurement Of Human Polyvalent Immunoglobulins: A Tender Analysis In Europe

**DOI:** 10.1017/S0266462325101803

**Published:** 2025-12-29

**Authors:** Jonathan Galduf, Carla Bellver, Laurie Marlow, Jie Shen, Nicholas Redman

## Abstract

**Introduction:**

Human polyvalent immunoglobulins (Igs) are plasma-derived medicinal products that are used to treat a range of conditions, including primary and secondary immunodeficiencies, hematologic diseases (immune thrombocytopenia), and neurological diseases. In Europe, public purchasers procure these essential medicines through tenders. Tenders across Europe are heterogeneous and differences in evaluation requirements at a regional level impact procurement decisions and, therefore, access to Igs.

**Methods:**

The aim of this research was to review tender requirements in European countries and provide insights into the regional variations in tender requirements for Igs. Data on tender criteria and procurement decisions for 27 countries in Europe were collected from documents available in the Tenders Electronic Daily archive and from stakeholders through interviews and questionnaires. A total of 1,160 tender documents were processed. The outcomes of tenders were assessed by market according to the importance of price, quality (shelf life and concentration), supply (delivery time), and sustainability (carbon footprint).

**Results:**

Many European markets procured at least three intravenous Igs and at least two subcutaneous Igs. Price was the only criterion evaluated for 945 (81.5%) of the tender documents evaluated. Price and quality criteria were both considered in 215 (18.5%) of the tender documents evaluated. Across Europe, the average weights of price and quality were 69.3 percent and 46.6 percent, respectively, suggesting that both factors affect decision-making. There was an upward trend in the influence of sustainability criteria in some markets. Supply criteria and technical requirements were considered to have a lower impact overall than quality or cost.

**Conclusions:**

In Europe, the price of Igs had the highest impact on procurement decisions, compared with quality, supply, and environmental requirements. However, many markets are procuring Igs from multiple suppliers, which may be a strategy to minimize potential supply shortages. Suppliers should therefore not underestimate the impact that other criteria, such as quality and consistent supply, can have on procurement decisions.

